# Alcohol as an independent risk factor for obstructive sleep apnea

**DOI:** 10.1007/s11845-021-02671-7

**Published:** 2021-06-10

**Authors:** Shanshan Yang, Xinhong Guo, Wei Liu, Yanhua Li, Yunxi Liu

**Affiliations:** 1 Department of Disease Prevention and Control, Chinese PLA General Hospital, The 1st Medical Center, Beijing, 100853 China; 2 Department of Cardiology, Chinese PLA General Hospital, The 1st Medical Center, Beijing, 1000853 China; 3 Chinese PLA General Hospital, Hospital Management Institute, Beijing, China

**Keywords:** Alcohol drinking, Case–control study, Conventional cardiovascular risk factors, Obstructive sleep apnea

## Abstract

**Background:**

To determine if alcohol consumption is a risk factor for obstructive sleep apnea (OSA) and nocturnal oxygen desaturation.

**Methods:**

This case–control study evaluated patients with confirmed OSA and a control group using polysomnography (PSG). Two doctors who have worked in this field for more than 5 years provided a blinded interpretation of the patients’ monitoring results. Logistic regression models were used to identify the odds ratio (OR) for alcohol consumption on OSA.

**Results:**

A total of 793 patients were enrolled in this study. Compared with those who did not consume alcohol, those consuming alcohol had a higher risk of OSA (OR 2.03, 95% CI 1.30–3.17) after adjustment. Regarding the risk of OSA after adjusting for former drinkers and current ones, the ORs were 1.96 (95% CI 1.19–3.22) and 2.22 (95% CI 1.06–4.63), respectively. And the P for trend = 0.002. The β of former drinkers and the current ones were 3.448 and 4.560 after adjustment; P for trend was 0.006. The relationship may have gender difference, and alcohol consumption was associated with AHI in female significantly (β = 10.190 and 15.395 for former and current drinkers, respectively, in females after adjustment, P for trend = 0.002).

**Conclusions:**

In this study, we found that alcohol consumption was an independent risk factor of OSA and OSA with hypoxia, and alcohol consumption was related to AHI significantly after adjustment, especially in female. In order to reduce the risk and severity of OSA, it is suggested that people should avoid drinking, and drinkers should abstain from drinking.

**Supplementary Information:**

The online version contains supplementary material available at 10.1007/s11845-021-02671-7.

## Introduction

Obstructive sleep apnea (OSA) is characterized by repetitive episodes of complete and partial obstructions of the upper airway during sleep. Previous studies [1] reported a prevalence of OSA in the general population of 2–10%, and more men were affected than women, but the prevalence of OSA was as high as 50% in patients with CHD and in those with metabolic syndrome [2]. In recent years, the prevalence of OSA has shown an increasing trend. OSA patients who experience hypoxia during sleep and are not treated for a long time may experience a variety of serious cardiovascular and cerebrovascular diseases, and even sudden death at night [3, 4]. A recent study by Franklin et al. [5] showed that OSA was present in 22% of male patients and 17% of female patients; women with OSA were more likely to develop hypertension, obesity, and other cardiovascular and cerebrovascular diseases that are age-related; and women with OSA may have clinical manifestations that differ from those of men, making the diagnosis more difficult. Obesity is a major risk factor for snoring and sleep apnea, but Franklin et al. reported that 39% of patients with OSA were of normal weight. Age and obesity are recognized risk factors for OSA. Other risk factors, such as the relationship between smoking and drinking, and sleep apnea remain contradictory. Foreign studies suggest that smoking and drinking are not fixed risk factors for OSA [6]. There are contradictory views regarding the relationship between alcohol consumption and OSA. Some studies [7] suggest that alcohol consumption increases the occurrence of hypoxia and apnea during sleep apnea episodes, but there are also some studies that have not found an increased risk for OSA of alcohol consumption [8, 9].

Many people develop OSA after drinking alcohol, particularly before sleep, and there is a high rate of alcohol consumption in China [10], but we still lack studies evaluating the correlation between alcohol consumption and OSA. And there is lack of information about alcohol consumption and OSA in Chinese populations especially; thus, this study will focus on the relationship between alcohol consumption and OSA in patients in China for further discussion.

## Materials and methods

### Study population

This case–control study involved inpatients with suspected OSA admitted from August 2014 to December 2017 to a large Chinese tertiary hospital. The inclusion criteria were (1) inpatients with suspected OSA and (2) inpatients who agreed to have overnight polysomnography (PSG) examination and were able to tolerate it. The exclusion criteria were those (1) could not be reached and (2) did not cooperate with the investigators. The patients were divided into definite OSA or control groups according to their sleep respiratory monitoring results.

The diagnostic criteria for OSA [11] were as follows: a polysomnographic study with more than 30 episodes of apnea–hypopnea during sleep 7 h per night or an apnea–hypopnea index (AHI) of ≥ 5 events/hour.

According to these test results, sleep apnea was either diagnosed or excluded. The patients signed voluntary consent to participate in the sleep respiratory monitoring, which was performed in our hospital respiratory sleep monitoring center from approximately 22:00–22:30 on the first day to the next day at about 5:00 the length of the monitoring time was 7 h. Two doctors who have worked in this field for more than 5 years provided a blinded interpretation of the patients’ monitoring results. The other related data were evaluated by a clinical doctor who was unaware of the patients’ sleep monitoring results to reduce bias.

All subjects underwent overnight polysomnography (PSG). Detailed records of the sleep respiratory monitoring test results and patient information collected during hospitalization were reliable. The demographic data and accompanying disease information, including age, gender, smoking status, alcohol consumption, height, weight, echocardiographic findings, and other medical conditions, were collected by a trained primary nurse. Age, gender, BMI, coronary heart disease (CHD), and stroke were adjusted in multivariate regression analysis. BMI was calculated as weight (kg)/standing height (m)^2^. CHD and stroke were confirmed by the attending physicians of the Cardiology Department of the large Chinese tertiary hospital according to WHO MONICA diagnostic criteria [12]. As a measure to ensure the accuracy of the information, the patient’s answers of the drinking information will be confirmed again by the patient and their relatives.

Alcohol consumption was defined as drinking at least one glass of wine within the past 30 days. According to the World Health Organization (WHO) definition [13], a glass of wine refers to 1/2 a bottle of beer, 2.5 glasses of wines or fruit wine, or 0.8 glasses of wine (1 glass is equal to 50 g).

### Statistical analysis

IBM SPSS Statistics for Windows, version 24.0, was used for the data analyses. The significance level for all tests was set at a two-tailed α value of 0.05. The differences in the means and proportions were evaluated using Student’s t- and chi-square tests, respectively. Logistic regression models were used to identify the association between alcohol consumption on OSA.

### Ethical considerations

This study was conducted in accordance with the Declaration of Helsinki and was approved by the Medical Ethics Committee of the Chinese PLA General Hospital. All participants provided written informed consent before joining the study.

## Results

A total of 793 patients were enrolled in this study, including 688 OSA patients and 106 control subjects. The average age was 57.4 ± 12.7 years (range 16–90 years). The average ages of those who with and without OSA were 57.7 ± 12.4 years (range 16–88 years) and 55.2 ± 14.1 years (range 25–90 years), respectively. There were no significant differences in age, coronary heart disease, stroke history, and body mass index between OSA patients and the control group. There were only differences in the sex ratio and alcohol consumption (*P* < 0.05, Table 1).

**Table 1:** Basline characteristics of participants

	*N* (%)	OSA		
	*n* = 794	No. (*n* = 108)	Yes (*n* = 688)	*P*
Gender				< 0.001
Male	614 (77.4)	41 (38.7)	138 (20.1)	
Female	179 (22.6)	65 (61.3)	550 (79.9)	
Age(years)				0.257
18–45	124 (15.6)	20 (18.9)	104 (15.1)	
46–64	472 (59.5)	66 (62.3)	407 (59.2)	
≥ 65	197 (24.8)	20 (18.9)	177 (25.7)	
Coronary heart disease				0.214
Yes	359 (45.3)	64 (60.4)	371 (53.9)	
No	434 (54.7)	42 (39.6)	317 (46.1)	
Stroke				0.244
Yes	103 (13.0)	96 (90.6)	595 (86.5)	
No	690 (87.0)	10 (9.4)	93 (13.5)	
BMI				0.222
< 18.5	1 (0.9)	2 (0.3)	0 (0.0)	
18.5–23.9	16 (15.1)	71 (10.4)	22 (6.0)	
24–27.9	46 (43.4)	276 (40.4)	157 (43.1)	
≥ 28	43 (40.6)	334 (48.9)	185 (50.8)	
Drinker				0.005
Yes	365 (46.0)	35 (33.3)	330 (48.0)	
No	428 (54.0)	70 (66.7)	358 (52.0)	

In the logistic regression, we found that, compared with those who did not consume alcohol, those consuming alcohol had a higher risk of OSA (OR 2.03, 95% CI 1.30–3.17) after adjusting for age, BMI, coronary heart disease, and stroke. Regarding the risk of OSA after adjusting for former drinkers and current ones, the ORs were 1.96 (95% CI 1.19–3.22, *P* < 0.05) and 2.22 (95% CI 1.06–4.63, *P* < 0.05), respectively. And the P for trend = 0.002 (Table 2). And we also found that, compared with those who did not consume alcohol, those who did had a higher risk of OSA and hypoxemia (OR 2.04, 95% CI 1.36–3.08) after adjusting for age, BMI, coronary heart disease, and stroke. Regarding the risk of OSA and hypoxemia after adjusting for former drinkers and current ones, the ORs were similar with ORs for OSA, and the P for trend = 0.001 (Table 3).

**Table 2:** Odds ratios (95% CIs) of OSA for alcohol use in participants

		*N* (%)	Model A	Model B	Model C
			OR (95% CI)	OR (95% CI)	OR (95% CI)
Alcohol use	None (reference)	358 (83.6)	1	1	1
	Yes	330 (90.4)	1.86 (1.21–2.87)	2.09 (1.34–3.25)	2.03 (1.30–3.17)
	P		0.005	0.001	0.002
Alcohol exposure group	None (reference)	358 (83.6)	1	1	1
	Former	231 (89.9)	1.75 (1.08–2.83)	2.01 (1.23–3.30)	1.96 (1.19–3.22)
	Current	99 (91.7)	2.18 (1.05–4.51)	2.28 (1.10–4.73)	2.22 (1.06–4.63)
	P for trend		0.005	0.001	0.002

Model A: crude model, model B: adjusted for age, model C: adjusted for age, BMI, coronary heart disease, and stroke

**Table 3:** Odds ratios (95% CIs) of OSA and hypoxemia for alcohol use in participants

		N (%)	Model A	Model B	Model C
			OR (95% CI)	OR (95% CI)	OR (95% CI)
Alcohol use	None (reference)	339 (79.2)	1	1	1
	Yes	321 (87.9)	1.93 (1.31–2.86)	2.13 (1.42–3.18)	2.04 (1.36–3.08)
	P		0.001	< 0.001	0.001
Alcohol exposure group	None (reference)	339 (79.2)	1	1	1
	Former	226 (87.9)	1.93 (1.24–3.00)	2.18 (1.38–3.43)	2.09 (1.32–3.33)
	Current	95 (88.0)	1.94 (1.04–3.63)	2.02 (1.08–3.78)	1.93 (1.02–3.64)
	P for trend		0.001	< 0.001	0.001

Model A: crude model, model B: adjusted for age, model C: adjusted for age, BMI, coronary heart disease, and stroke

AHI is the most objective measure of the severity of OSA, so we did the linear regression models to analyze the relationship between alcohol consumption and the AHI in the participants. The result showed that the β of former drinkers and the current ones were 3.448 and 4.560 after adjusted for age, BMI, coronary heart disease, and stroke respectively; P for trend was 0.006 (Table 4). And the relationship may have gender difference, alcohol consumption was associated with AHI in female significantly (β = 10.190 and 15.395 for former and current drinkers respectively in females after adjustment, P for trend = 0.002; Table 4).

**Table 4:** Effect of alcohol use on AHI in participants and in different genders

	Model A						Model B					Model C					
		95% CI						95% CI						95% CI			
	ß	Lower	Upper	Standard ß	P for trend	ß		Lower	Upper	Standard ß	P for trend		ß	Lower	Upper	Standard ß	P for trend
None (reference)					0.003						0.002						0.006
Former	3.383	0.429	6.336	0.083		3.779		0.735	6.823	0.093			3.448	0.418	6.478	0.085	
Current	5.134	1.112	9.157	0.093		5.259		1.230	9.288	0.095			4.560	0.551	8.568	0.082	
Male																	
None (reference)					0.422						0.388						0.621
Former	0.720	-2.600	4.041	0.018		0.885		− 2.520	4.289	0.023			0.481	− 2.901	3.864	0.012	
Current	1.931	-2.528	6.390	0.097		1.979		− 2.489	6.446	0.038			1.146	− 3.283	5.575	0.022	
Female																	
None (reference)					0.003						0.003						0.002
Former	9.657	− 2.422	21.737	0.117		9.651		− 2.295	21.598	0.117			10.190	− 1.936	22.317	0.123	
Current	14.914	3.922	25.906	0.198		14.481		3.604	25.359	0.192			15.395	4.405	26.385	0.205	

Model A: crude model, model B: adjusted for age, model C: adjusted for age, BMI, coronary heart disease, and stroke

We also did the logistic regression analysis of Tables 2 and 3 in different gender, and the results were similar (Tables S1 and S2). However, the ORs were not significant in both male and female, and this may due to the reason that few women were drinkers in China for the cultural impact.

## Discussion

In this study, we found that alcohol consumption was an independent risk factor of OSA and OSA with hypoxia, and current drinks had a higher risk of OSA and OSA with hypoxia than former ones. Drinking cessation reduced the increased OSA risk associated with alcohol consumption, but previous alcohol exposure still increases the OSA risk when compared with people who never drink. Alcohol consumption was related to AHI significantly after adjustment, especially in female.

Previous studies showed that the risk factors of OSA are complex; besides obesity, gender, and upper airway anatomical abnormalities, alcohol consumption is an important risk factor [14]. A study of teenagers in Hong Kong showed that drinking not only aggravates sleep difficulties and insomnia, but also makes snoring more pronounced among drinkers [15]. The results of this study suggested that AHI was positively correlated with alcohol consumption, and LSaO2 was negatively correlated with alcohol consumption, which is consistent with the results of Pan Y et al. [16]. And we firstly showed that there was gender difference in the effect of alcohol on OSA and AHI in Chinese population.

The mechanism of OSA induced by alcohol consumption may be related to the collapse of upper airway caused by alcohol consumption [17]. Further, experimental studies have shown that chronic alcohol intake can lead to alveolar type 2 cell damage and alveolar surface glutathione reduction [18]. In ewes exposed to alcohol for a long time, chronic lung injury of fetus, decrease of type 2 cells and SPA on alveolar surface, and decrease of expression of alveolar surface protein A and C by one-third [19, 20], these may be the mechanisms of OSA induced by alcohol consumption, but the mechanism of gender differences remains to be further explored.

This study has several limitations. Firstly, this study is a case–control study with inherent memory bias, but we tried to minimize the memory bias by confirming the information of drinking habits to the family members when the progress of the information collection. Secondly, the population in this study is the hospitalized population of a high-level hospital, which is not well represented, and the extrapolation of conclusions needs to be cautious.

## Conclusion

In this study, we found that alcohol consumption was an independent risk factor of OSA and OSA with hypoxia, and alcohol consumption was related with AHI significantly after adjustment, especially in female. In order to reduce the risk and severity of OSA, it is suggested that people should avoid drinking, and drinkers should abstain from drinking.

## Supplementary Information

Below is the link to the electronic supplementary material.

Supplementary file1 (DOC 121 KB)

## Data Availability

The datasets used and/or analyzed during the current study available from the corresponding author on reasonable request.

## Data availability

The datasets used to support this study are not freelyavailable in view of participants’ privacy protection.
