# Supplementary material for: Alcohol as an independent risk factor for obstructive sleep apnea
Source: Ir J Med Sci. 2021 Jun 10;191(3):1325–30. doi: 10.1007/s11845-021-02671-7 (PMC9135842; doi:10.1007/s11845-021-02671-7)
Supplement: Supplementary file 1 — Supplementary file1 (DOC 121 KB) [file 11845_2021_2671_MOESM1_ESM.doc]

| Table1 Basline characteristics of participants | | | | |
| --- | --- | --- | --- | --- |
|  | N(%) | OSA | | |
|  | n=794 | No (n=108) | Yes (n=688) | P |
| Gender |  |  |  | <0.001 |
| Male | 614 (77.4) | 41 (38.7) | 138 (20.1) |  |
| Female | 179 (22.6) | 65 (61.3) | 550 (79.9) |  |
| Age(years) |  |  |  | 0.257 |
| 18-45 | 124 (15.6) | 20 (18.9) | 104 (15.1) |  |
| 46-64 | 472 (59.5) | 66 (62.3) | 407 (59.2) |  |
| ≥65 | 197 (24.8) | 20 (18.9) | 177 (25.7) |  |
| Coronary heart disease |  |  |  | 0.214 |
| Yes | 359 (45.3) | 64 (60.4) | 371 (53.9) |  |
| No | 434 (54.7) | 42 (39.6) | 317 (46.1) |  |
| Stroke |  |  |  | 0.244 |
| Yes | 103 (13.0) | 96 (90.6) | 595 (86.5) |  |
| No | 690 (87.0) | 10 (9.4) | 93 (13.5) |  |
| BMI |  |  |  | 0.222 |
| <18.5 | 1 (0.9) | 2 (0.3) | 0 (0.0) |  |
| 18.5-23.9 | 16 (15.1) | 71 (10.4) | 22 (6.0) |  |
| 24-27.9 | 46 (43.4) | 276 (40.4) | 157 (43.1) |  |
| ≥28 | 43 (40.6) | 334 (48.9) | 185 (50.8) |  |
| Drinker |  |  |  | 0.005 |
| Yes | 365 (46.0) | 35 (33.3) | 330 (48.0) |  |
| No | 428 (54.0) | 70 (66.7) | 358 (52.0) |  |

**Table 2.**Odds ratios (95% CIs) of OSA for alcohol use in participants

|  | | | | | |
| --- | --- | --- | --- | --- | --- |
|  |  | N (%) | Model A | Model B | Model C |
|  |  |  | OR (95% CI) | OR (95% CI) | OR (95% CI) |
| Alcohol use | |  |  |  |  |
|  | None (reference) | 358 (83.6) | 1 | 1 | 1 |
|  | Yes | 330 (90.4) | **1.86 (1.21-2.87)** | **2.09 (1.34-3.25)** | **2.03 (1.30-3.17)** |
|  | P |  | 0.005 | 0.001 | 0.002 |
| Alcohol exposure group |  |  |  |  |  |
|  | None (reference) | 358 (83.6) | 1 | 1 | 1 |
|  | Former | 231 (89.9) | **1.75 (1.08-2.83)** | **2.01 (1.23-3.30)** | **1.96 (1.19-3.22)** |
|  | Current | 99 (91.7) | **2.18 (1.05-4.51)** | **2.28 (1.10-4.73)** | **2.22 (1.06-4.63)** |
|  | P for trend |  | 0.005 | 0.001 | 0.002 |
| Model A: Crude model  Model B: Adjusted for age  Model C: Adjusted for age, BMI, coronary heart disease, and stroke | | | | | |

**Table 3.** Odds ratios (95% CIs) of OSA and hypoxemia for alcohol use in participants

|  | | | | | |
| --- | --- | --- | --- | --- | --- |
|  |  | N (%) | Model A | Model B | Model C |
|  |  |  | OR (95% CI) | OR (95% CI) | OR (95% CI) |
| Alcohol use | |  |  |  |  |
|  | None (reference) | 339 (79.2) | 1 | 1 | 1 |
|  | Yes | 321 (87.9) | **1.93 (1.31-2.86)** | **2.13 (1.42-3.18)** | **2.04 (1.36-3.08)** |
|  | P |  | 0.001 | <0.001 | 0.001 |
| Alcohol exposure group |  |  |  |  |  |
|  | None (reference) | 339 (79.2) | 1 | 1 | 1 |
|  | Former | 226 (87.9) | **1.93 (1.24-3.00)** | **2.18 (1.38-3.43)** | **2.09 (1.32-3.33)** |
|  | Current | 95 (88.0) | **1.94 (1.04-3.63)** | **2.02 (1.08-3.78)** | **1.93 (1.02-3.64)** |
|  | P for trend |  | 0.001 | <0.001 | 0.001 |

Model A: Crude model

Model B: Adjusted for age

Model C: Adjusted for age, BMI, coronary heart disease, and stroke

Table 4.Effect of alcohol use on AHI in participants and in different genders

|  | | | | | | | | | | | | | | | | | | |
| --- | --- | --- | --- | --- | --- | --- | --- | --- | --- | --- | --- | --- | --- | --- | --- | --- | --- | --- |
|  | Model A | | | |  | | | Model B | | | | |  | Model C | | | | |
|  |  | 95%CI | |  |  |  | 95%CI | | |  |  |  | | | 95%CI | |  |  |
|  | ß | lower | upper | Standard ß | P for trend | ß | lower | | upper | Standard ß | P for trend | ß | | | lower | upper | Standard ß | P for trend |
| None (reference) |  |  |  |  | 0.003 |  |  | |  |  | 0.002 |  | | |  |  |  | 0.006 |
| Former | 3.383 | 0.429 | 6.336 | 0.083 |  | 3.779 | 0.735 | | 6.823 | 0.093 |  | 3.448 | | | 0.418 | 6.478 | 0.085 |  |
| Current | 5.134 | 1.112 | 9.157 | 0.093 |  | 5.259 | 1.230 | | 9.288 | 0.095 |  | 4.560 | | | 0.551 | 8.568 | 0.082 |  |
| **Male** |  |  |  |  |  |  |  | |  |  |  |  | | |  |  |  |  |
| None (reference) |  |  |  |  | 0.422 |  |  | |  |  | 0.388 |  | | |  |  |  | 0.621 |
| Former | 0.720 | -2.600 | 4.041 | 0.018 |  | 0.885 | -2.520 | | 4.289 | 0.023 |  | 0.481 | | | -2.901 | 3.864 | 0.012 |  |
| Current | 1.931 | -2.528 | 6.390 | 0.097 |  | 1.979 | -2.489 | | 6.446 | 0.038 |  | 1.146 | | | -3.283 | 5.575 | 0.022 |  |
| **Female** |  |  |  |  |  |  |  | |  |  |  |  | | |  |  |  |  |
| None (reference) |  |  |  |  | 0.003 |  |  | |  |  | 0.003 |  | | |  |  |  | 0.002 |
| Former | 9.657 | -2.422 | 21.737 | 0.117 |  | 9.651 | -2.295 | | 21.598 | 0.117 |  | 10.190 | | | -1.936 | 22.317 | 0.123 |  |
| Current | 14.914 | 3.922 | 25.906 | 0.198 |  | 14.481 | 3.604 | | 25.359 | 0.192 |  | 15.395 | | | 4.405 | 26.385 | 0.205 |  |

Model A: Crude model

Model B: Adjusted for age

Model C: Adjusted for age, BMI, coronary heart disease, and stroke

**Table S1.**Odds ratios (95% CIs) of OSA for alcohol use in different genders

|  | | | | |
| --- | --- | --- | --- | --- |
|  | N (%) | Model A | Model B | Model C |
|  |  | OR (95% CI) | OR (95% CI) | OR (95% CI) |
| Male |  |  |  |  |
| None (reference) | 238 (88.5) | 1 | 1 | 1 |
| Former | 223 (89.9) | 1.17 (0.67-2.04) | 1.25 (0.71-2.22) | 1.23 (0.69-2.18) |
| Current | 89 (91.8) | 1.46 (0.65-3.30) | 1.49 (0.66-3.37) | 1.44 (0.64-3.28) |
| P for trend |  | 0.358 | 0.279 | 0.329 |
| Female |  |  |  |  |
| None (reference) | 120 (75.5) | 1 | 1 | 1 |
| Former | 8 (88.9) | 2.64 (0.32-21.81) | 3.12 (0.34-28.34) | 3.84 (0.37-39.53) |
| Current | 10 (90.9) | 3.31 (0.41-21.65) | 2.88 (0.35-23.60) | 2.88 (0.34-24.14) |
| P for trend |  | 0.160 | 0.179 | 0.165 |

Model A: Crude model

Model B: Adjusted for age

Model C: Adjusted for age, BMI, coronary heart disease, and stroke

**Table S2.** Odds ratios (95% CIs) of OSA and hypoxemia for alcohol use in different genders

|  | | | | |
| --- | --- | --- | --- | --- |
|  | N (%) | Model A | Model B | Model C |
|  |  | OR (95% CI) | OR (95% CI) | OR (95% CI) |
| Male |  |  |  |  |
| None (reference) | 225 (83.6) | 1 | 1 | 1 |
| Former | 219 (88.3) | 1.48 (0.90-2.46) | 1.55 (0.93-2.60) | 1.49 (0.88-2.52) |
| Current | 85 (87.6) | 1.40 (0.70-2.77) | 1.42 (0.71-2.81) | 1.36 (0.68-2.72) |
| P for trend |  | 0.134 | 0.111 | 0.162 |
| Female |  |  |  |  |
| None (reference) | 114 (71.7) | 1 | 1 | 1 |
| Former | 7 (77.8) | 1.41 (0.28-7.03) | 1.55 (0.28-8.66) | 2.14 (0.33-13.82) |
| Current | 10 (90.9) | 4.02 (0.50-32.31) | 3.55 (0.43-28.96) | 3.21 (0.38-27.00) |
| P for trend |  | 0.175 | 0.201 | 0.188 |

Model A: Crude model

Model B: Adjusted for age

Model C: Adjusted for age, BMI, coronary heart disease, and stroke
